# Smoking cessation by combined medication and counselling: a feasibility study in lung cancer patients

**DOI:** 10.1186/s12890-022-02048-1

**Published:** 2022-06-27

**Authors:** Christian Reinhardt, Markus Harden, Christoph Herrmann-Lingen, Achim Rittmeyer, Stefan Andreas

**Affiliations:** 1 Lungenfachklinik Immenhausen, Immenhausen, Germany; 2 Institute of Medical Statistics, University Medical Center Göttingen, Göttingen, Germany; 3 Department of Psychosomatic Medicine and Psychotherapy, University Medical Center Göttingen, Göttingen, Germany; 4 Department of Cardiology and Pneumology, Member of the German Center for Lung Research (DZL), University Medical Center Göttingen, Robert-Koch-Str. 40., Göttingen, Germany

**Keywords:** Smoking cessation, Lung cancer, Pharmacotherapy, Varenicline, Real life setting

## Abstract

**Purpose:**

Smoking cessation in patients with diagnosed lung cancer has positive effects on cancer therapy and overall prognosis. Despite this, knowledge on smoking cessation in lung cancer patients is sparse.

**Methods:**

This is an observational single centre, 12-week, prospective, single-arm trial at a tertiary lung cancer centre. Responsive patients were enrolled following confirmed lung cancer diagnosis. Smoking cessation intervention included counselling as well as pharmacotherapy. The primary endpoint was the point prevalence abstinence rate at week 12 based on biochemical verification. Secondary endpoints were the abstinence rate at week 26, quality of life and side effects.

**Results:**

80 patients were enrolled. Mean age was 62.6 ± 7.9 years. Most patients (63%) were treated with chemotherapy or radiochemotherapy. 39 patients used nicotine replacement therapy, 35 varenicline whereas six patients did not use pharmacotherapy. During the study period 13 patients died. Data were available in 72 patients after 12 weeks and 57 patients at week 24. Point prevalence abstinence rates were 37.5% (95% CI 26.4–49.7%) at week 12 and 32.8% (95% CI 21.8–45.4%) at week 26, respectively. Quality of life and side effects were not significantly affected by pharmacotherapy.

**Conclusion:**

In conclusion, our results suggest that smoking cessation is feasible in patients with newly diagnosed lung cancer. The observed abstinence rate is comparable to other patient cohorts. Furthermore, pharmacotherapy in addition to cancer therapy was safe and did not show novel side effects in these seriously ill patients. Thus, smoking cessation should be an integral part of lung cancer treatment.

*Trial registration* The study was conducted in accordance with good clinical practice standards (GCP) and approved by the local ethics committee (16/3/14), the European PAS registry (EUPAS8748) and the German BfArM (NIS-Studien-Nr. 5508). All patients provided written informed consent before study enrollment.

**Supplementary Information:**

The online version contains supplementary material available at 10.1186/s12890-022-02048-1.

## Background

Tobacco smoking increases mutational burden and quitting fosters replenishment of the bronchial epithelium with cells that have avoided tobacco mutagenesis [1]. Thus, not surprisingly, smoking cessation is not only paramount in the prevention but also in the treatment of lung cancer. Indeed, quitting smoking clearly improves lung cancer survival. Recent studies show that smoking cessation after the diagnosis of lung cancer had an influence on overall prognosis [2, 3], effectiveness of chemotherapy [4] and on morbidity after curative surgery [5, 6]. Smoking cessation has therefore become a part of current treatment guidelines for lung cancer [7, 8]. The majority of lung cancer patients is willing to stop smoking. Due to their long smoking history and high nicotine dependence, the ability to reach this aim is low. Recent data show a still high smoking rate in smoking-related cancer survivors [7].

Concerning hospitalized patients mainly with cardiovascular disease a meta-analysis demonstrated that high-intensity behavioral interventions (preferable with pharmacologic therapy) beginning during a hospital stay promote smoking cessation [9, 10].

Despite the above mentioned, clinicians often hesitate to actively encourage patients to quit smoking once lung cancer is diagnosed. Reasons for this might be ignorance of the clinical benefits and medical options for smoking cessation, lack of time for smoking cessation counselling, and fear of side effects of available drugs for smoking cessation during cancer treatment [11].

Until now, data on smoking cessation in lung cancer patients are sparse. In a metaanalysis there was insufficient evidence to determine whether smoking cessation interventions are effective for people with lung cancer [8]. In a recent large retrospective study investigating patients with current cancer an intensive cessation treatment resulted in an overall self reported abstinence rate of 45% at 3 months. The subgroup of patients with lung cancer demonstrated similar results as compared to the overall group [12]. To investigate whether smoking cessation could be implemented in the daily routine of a lung cancer centre we conducted this single arm, uncontrolled feasibility study. The primary endpoint was the point prevalence abstinence rate at week 12 based on biochemical verification. Quality of life and side effects were also prospectively evaluated.

## Methods

### Study design and outcome

This is an observational, single center, 12-week, prospective, single-arm trial of a smoking cessation intervention performed at a large university lung cancer centre. The primary endpoint was the point abstinence rate at week 12 based on biochemical verification. Secondary endpoints were the point abstinence rate at week 26, quality of life and abstinence phenomena as evaluated by questionnaire (see study procedures). Patients were enrolled after confirmed lung cancer diagnosis. Every patient with a new diagnosis of lung cancer was screened, unless the study team was not available.

Inclusion criteria were as follows:

- Newly diagnosed lung cancer stages I–IV within 14 days preceding study enrollment
- Current smoking or smoking up to 4 weeks before study enrollment
- Age ≥ 18 years
- Providing written informed consent

Exclusion criterion was severe comorbidity, making study participation unlikely.

The study was conducted in accordance with good clinical practice standards (GCP) and approved by the local ethics committee (Ethikkommission der Universitätsmedizin Göttingen, 16/3/14), the European PAS registry (EUPAS8748) and the Bundesinstitut für Arzneimittel und Medizinprodukte (BfArM) (NIS-Studien-Nr. 5508) before enrolment. All patients provided written informed consent before study related procedures.

### Study procedures

After enrollment, each patient received smoking cessation counselling by trained physicians or a social worker. Counselling was based on the technique of motivational interviewing. It also included counselling on pharmacotherapy. Following counselling, patients could decide whether they wanted to use pharmaceutical support or not. Pharmacotherapy offered was nicotine replacement therapy in form of patches and/or nicotine gums/lozenges according to the daily number of cigarettes smoked or varenicline for 12 weeks each. Patients received verbal and written instructions on how to use pharmacotherapy.

The intervention was conducted in accordance with the German procedure code (OPS) for in hospital smoking cessation (OPS 9-501 “Multimodale stationäre Behandlung zur Tabakentwöhnung” [13]. However, the duration of the intervention was at least 30 min and thus shorter as stated in the OPS. Physicians and the social worker were trained and certified according to the curriculum of the Bundesärztekammer. This curriculum comprises 28 h of structured learning in small groups and is constructed to address the four main active ingredients according to behavior change technique taxonomy [14].

At study entry, carbon monoxide (CO)-Concentration in exhaled air was measured using a Senko® BMC 2000 device (SENKO Co., Ltd.). CO-Concentration in exhaled air of more than 8 ppm was considered as current smoking.

Cigarette Dependence was measured by the Fagerström Test for Cigarette Dependence [15–17]. Quality of life was assessed at the beginning and week 12 by the EORTC QLQ C30 and [18] the lung-specific EORTC QLQ LC13 questionnaire [19] as well as the EQ VAS [18]. In addition, we assessed psychological distress by the Hospital Anxiety and Depression Scale (HADS) and abstinence phenomena by the latest version of the Mood and physical symptoms scale (MPSS) [20, 21].

**Fig. 1:**
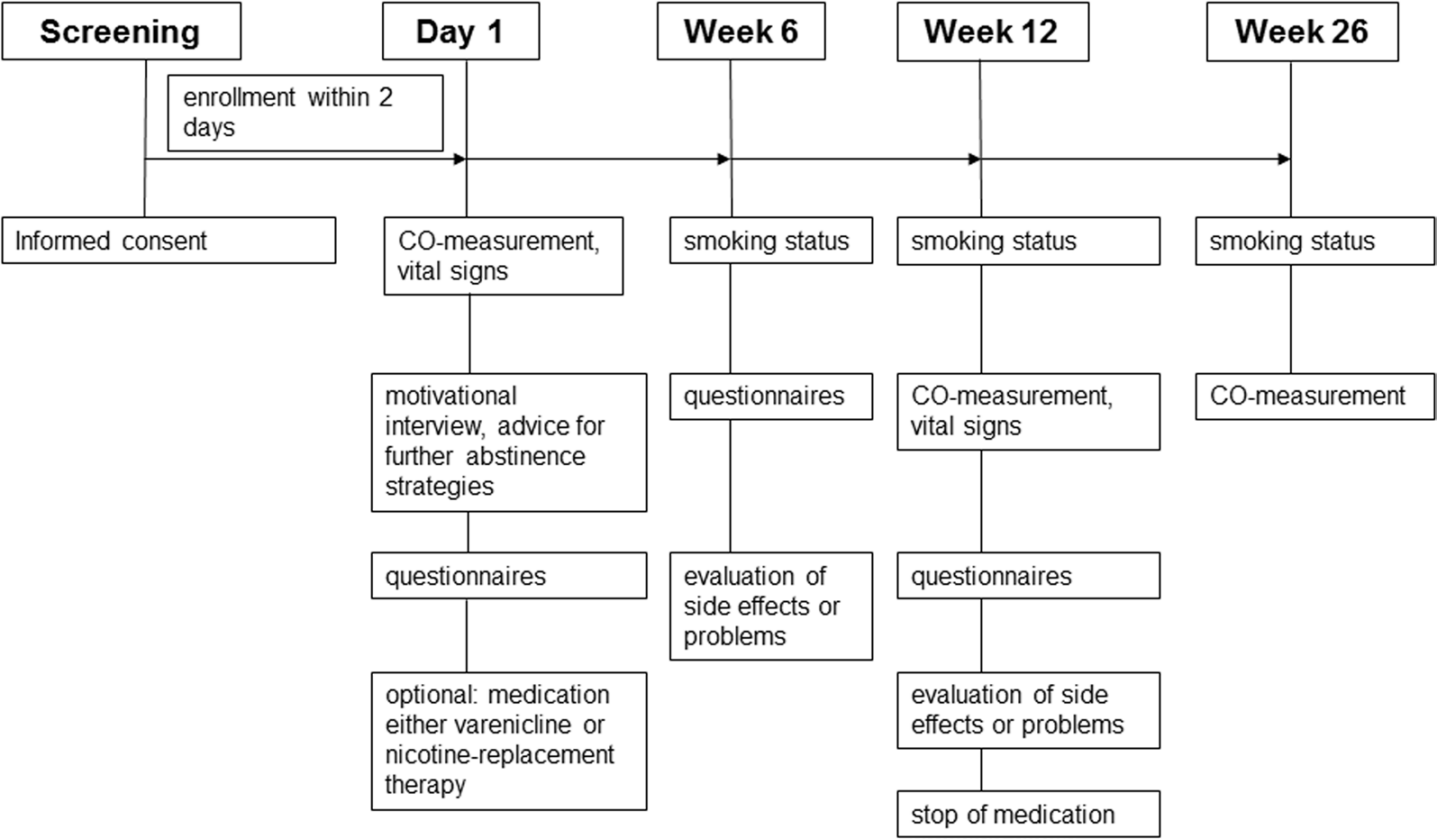
Study flow

After 6 weeks, patients were asked about their smoking status, withdrawal symptoms, the use of the medication (if chosen) and any side effects. After 12 weeks, they were also asked about quality of life and the CO concentration in exhaled air was measured again. After 26 weeks CO concentration was measured. Study flow and investigations are shown in Fig. 1.

### Statistical analysis

Baseline characteristics of analyzed patients are reported by summary statistics such as mean (standard deviation) for continuous data and frequencies (percentages) for categorical data overall and by intervention. Patients who withdrew their consent or were lost to follow up were counted as smokers according to the intention to treat protocol. Patients who died during the study period were censored after their last documented visit.

Tobacco abstinence was defined as CO concentration in exhaled air below or equal to 8 ppm. Missing values were considered as non-abstinent. The primary endpoint tobacco abstinence after 12 weeks was estimated with 95% Clopper–Pearson confidence intervals. Differences between the treatment groups, albeit not randomized, were tested using the *t*-test.

Since this was not a controlled efficacy study no formal power calculation was performed. Nevertheless, we planned that 80 patients could be enrolled in 2–3 years based on previous experience [22, 23]. With a 10% drop out rate 72 patients would be available for calculation of tobacco abstinence.

The funding source had no influence on the study design, the data analysis and interpretation of the study.

## Results

Study enrollment ran from March 2015 to June 2018. Baseline characteristics of the study population are shown in Table 1. Of 80 patients enrolled, 39 decided for nicotine replacement therapy, 35 for varenicline whereas six patients chose no pharmacotherapy.

**Table 1:** Patient baseline characteristics

	NRT^a^ (n = 39)	Varenicline (n = 35)	NM^b^ (n = 6)	Total (n = 80)
Sex				
Female	11 (28.2%)	11 (31.4%)	2 (33.3%)	24 (30%)
Male	28 (71.8%)	24 (68.6%)	4 (66.7%)	56 (70%)
Age (years)	62.9 (± 8.4)	62.6 (± 7.8)	59.5 (± 4.9)	62.6 (± 7.9)
BMI (kg/m^2^)	25.9 (± 4.8)	25.2 (± 4.8)	24.8 (± 5.3)	25.5 (± 4.8)
Score of Fagerstroem Test	4.7 (± 2.2) (13 missing)	4.9 (± 2.4) (2 missing)	4.0 (± 1.4) (4 missing)	4.8 (± 2.3) (19 missing)
PackYears	41.92 (± 18)	49.58 (± 27.51)	36.9 (± 9.6)	44.9 (± 22.5)
At least one attempt for smoking cessation	18 (46.2%)	18 (51.4%)	1 (16.7%)	37 (46.3%)
Current smoking at study entry	26 (66.7%)	31 (88.6%)	1 (16.7%)	58 (72.5%)
Quit smoking within 4 weeks before study entry	13 (33.3%)	4 (11.4%)	5 (83.3%)	22 (27.5%)
ECOG**				
0	7	7	2	16
1	25	25	2	52
2	1	0	0	1
Lung-Cancer Stage				
NSCLC I–II	7 (18%)	6 (17.1%)	1 (16.7%)	14 (17.5%)
NSCLC III–IV	17 (43.6%)	20 (57.1%)	2 (33.3%)	39 (49%)
SCLC	15 (38.5%)	9 (25.7%)	3 (50%)	27 (34%)
Lung-Cancer Therapy				
Surgery	10 (25.6%)	5 (14.3%)	1 (16.7%)	16 (20%)
Chemotherapy or radiochemotherapy	29 (74.4%)	29 (82.9%)	5 (83.3%)	63 (78.8%)
Radiotherapy	0	1 (2.9%)	0	1 (1.2%)

*Percent of Nicotine replacement group

**Eastern cooperative oncology group, in 11 patients no data available

^a^*NRT* nicotine replacement therapy, ^b^*NM* no medication group

Of the 80 patients, 66% had non-small cell lung cancer and 34% small cell lung cancer. During the study period 13 patients died (nine due to worsening of lung cancer, 1 due to myocardial infarction, 1 due to renal failure, 1 due to stroke, one patient committed suicide after receiving the message of cancer progress). Four patients were lost to follow up and 7 patients withdrew their consent. One patient was diagnosed as non-small cell lung cancer and received nicotine replacement therapy. A few days following study enrollment, the final diagnosis was urothelial cancer and the patient was subsequently excluded. Some patients responded to a call for visit 4 but not for visit 3. Thus, data are available of 72 patients after 12 weeks and of 67 patients at week 26.

Overall verified tobacco point abstinence rate for patients alive was 37.5% (95% CI 26.4–49.7%) at week 12 and 32.8% (95% CI 21.8–45.4%) at week 26, respectively. Point abstinence rates according to medical treatment are shown in Table 2.

**Table 2:** Point abstinence rates

	Week 12			Week 26		
	Smoking	Abstinent	Total	Smoking	Abstinent	Total
NRT^a^	27 (71.1%)	11 (28.9%)	38 (52.8%)	25 (69.4%)	11 (30.6%)	36 (53.7%)
Varenicline	16 (55.2%)	13 (44.8%)	29 (40.3%)	18 (66.7%)	9 (33.3%)	27 (40.3%)
NM^b^	2 (40%)	3 (60%)	5 (6.9%)	2 (50%)	2 (50%)	4 (6%)
Total	45 (62.5%)	27 (37.5%)	72 (100%)	45 (67.2%)	22 (32.8%)	67 (100%)

Abstinence rate at week 12 and 26

^a^*NRT* nicotine replacement therapy, ^b^*NM* no medication group

**Fig. 2:**
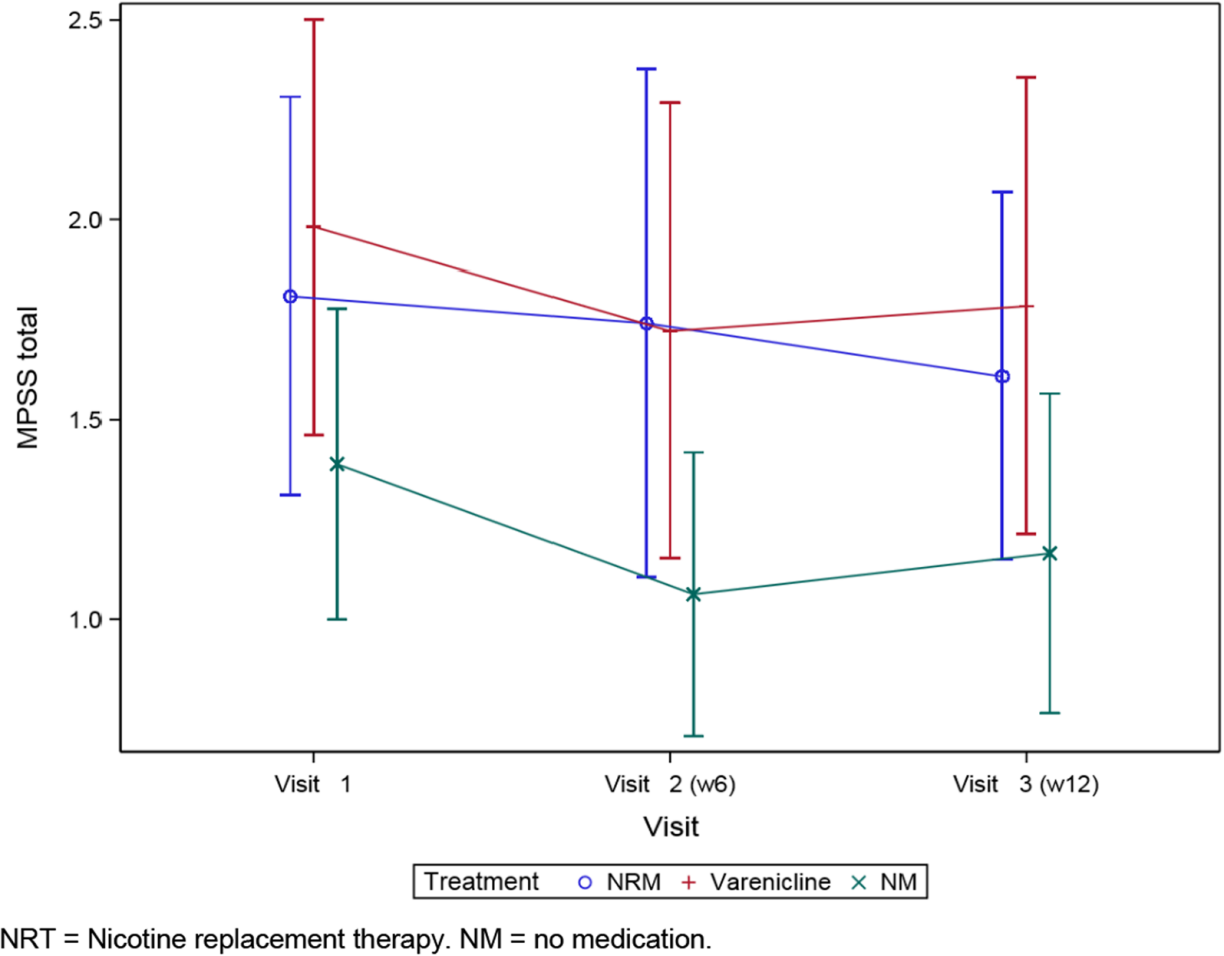
MPSS (± standard deviation) during treatment period. *NRT* Nicotine replacement therapy, *NM* no medication

*Withdrawal symptoms* evaluated by MPSS-Scores of the treatment groups are shown in Fig. 2. There was no increase in MPSS-Scores during the treatment period. There was a significant difference in withdrawal symptoms between patients in the no medication group and patients in the NRT or varenicline group. Patients in the no medication group showed significantly fewer withdrawal symptoms than others right from the beginning up to week 12. There were no significant differences in abstinence phenomena between the NRT and varenicline group (for details see Tables 3, 4). Most patients of the no medication group had already stopped smoking within four weeks before study entry (5 out of 6 patients) and showed a low Fagerström score.

**Table 3:** Mean EORTC-QLQ C30 3.0 subscale *nausea and vomiting* at day 1 and week 12

	Treatment	N	NMiss	Mean	STD	Mean (95% LCL)	Mean (95% UCL)
Day 1	NRT	37	2	5.9	11.3	2.1	9.6
	Varenicline	35	0	6.7	16.8	0.9	12.4
	NM	6	0	2.8	6.8	− 4.4	9.9
	Total	78	2	6.0	13.7	2.9	9.1
Week 12	NRT	23	16	24.6	27.5	12.8	36.5
	Varenicline	24	11	22.9	27.7	11.2	34.6
	NM	5	1	10.0	22.4	− 17.8	37.8
	Total	52	28	22.4	27.0	14.9	30.0

There was no significant difference between the groups

*NRT* Nicotine replacement therapy, *NM* no medication, *STD* standard deviation

**Table 4:** Mean EORTC-QLQ C30 3.0 subscale *insomnia* at day 1 and week 12

	Treatment	N	NMiss	Mean	STD	Mean (95% LCL)	Mean (95% UCL)
Day 1	NRT	36	3	30.6	33.2	19.3	41.8
	Varenicline	34	1	44.1	36.4	31.4	56.8
	NM	6	0	50.0	40.8	7.2	92.8
	Total	76	4	38.2	35.6	30.0	46.3
Week 12	NRT	23	16	40.6	36.2	24.9	56.2
	Varenicline	24	11	43.1	31.8	29.6	56.5
	NM	5	1	13.3	29.8	− 23.7	50.4
	Total	52	28	39.1	34.1	29.6	48.6

There was no significant difference between the groups

*NRT* Nicotine replacement therapy, *NM* no medication, *STD* standard deviation

**Fig. 3:**
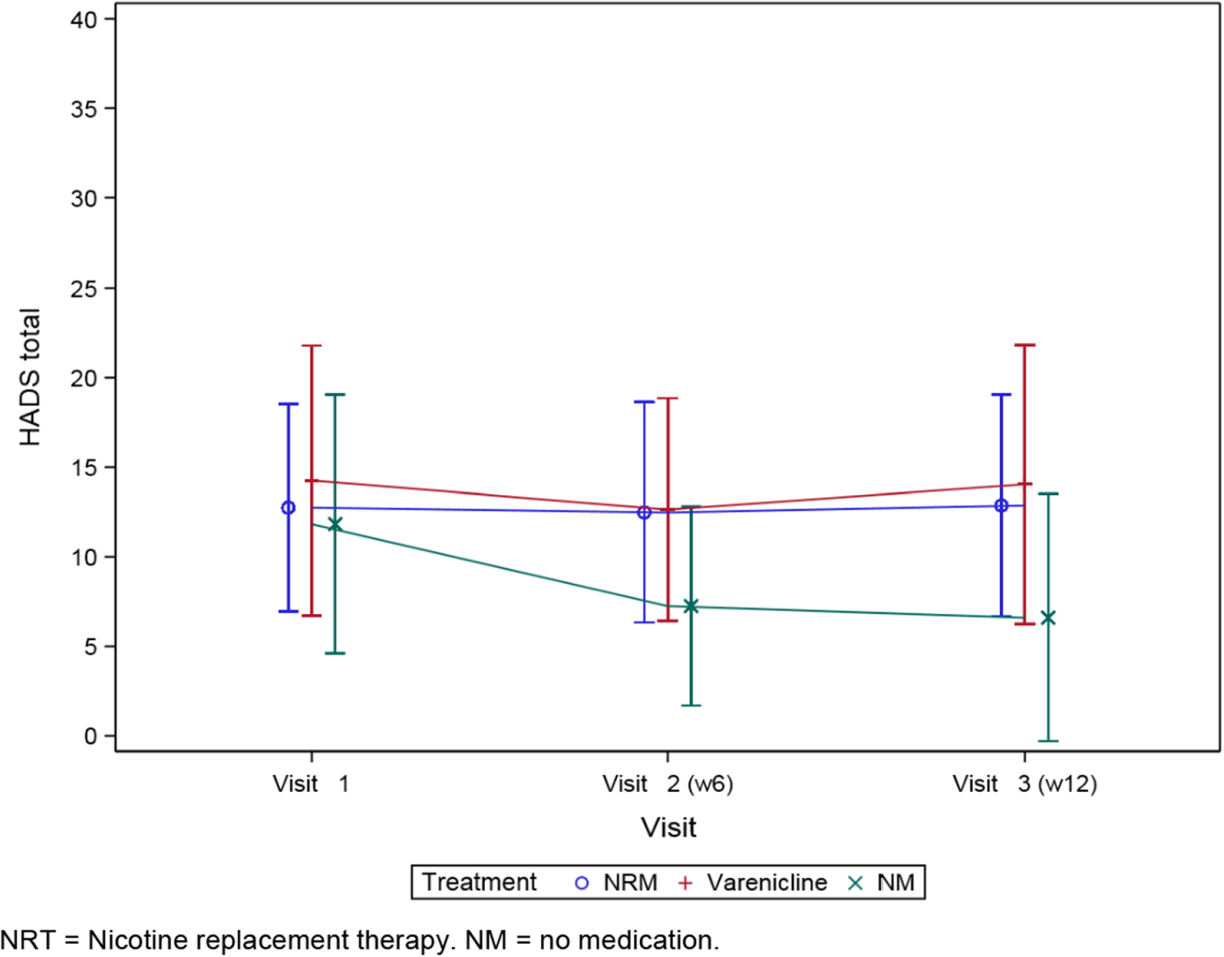
HADS (± standard deviation) during treatment period. *NRT* Nicotine replacement therapy. *NM* no medication

HADS scores for each treatment are shown in Fig. 3. There was no increase in HADS total score during the treatment period. There was a tendency to a difference in symptoms between patients in the no medication group and patients in the NRT or varenicline group at week 6 and week 12 (Additional file 1: HADS).

There was no group difference in EQ VAS, EORTC QLQ C30 and EORTC LC13 subscales. For further details, see Additional file 2: EORTC.

### Side effects

Each patient was asked at visit 2 (week 6) and visit 3 (week 12) if adverse events of medication occurred. Hospitalization due to lung cancer therapy or lung cancer therapy complications was not considered an adverse event as predefined in the study protocol.

#### Nicotine replacement therapy

Patients receiving nicotine replacement therapy were, amongst others, asked at visit 2 and 3 about skin irritation, flu-like symptoms and coughing due to the therapy. Furthermore, they were asked about neurological, cardiovascular and endocrinological symptoms. None of the 39 patients receiving nicotine replacement therapy reported any of these symptoms at visit 2 and visit 3, respectively.

One patient suffered a myocardial infarction four weeks after visit 3 and died of cardiogenic shock two days later. At that time, the patient had already stopped nicotine replacement therapy.

#### Varenicline

Patients who received varenicline were, amongst others, questioned about psychological effects such as abnormal dreams, insomnia, anxiety, and hallucinations. Furthermore, they were asked about gastrointestinal side effects such as nausea, vomiting, obstipation, and chest pain. Six patients reported nausea at week 6, 3 also reported vomiting. All these patients stopped varenicline without further symptoms. One patient switched to nicotine replacement therapy. Of note, all patients reporting nausea received chemotherapy or radiochemotherapy, respectively. When comparing EORTC QLQ C30 subscales nausea and vomiting as well as fatigue and insomnia, there was no difference at week 12 between treatment groups (Additional file 2: EORTC).

In one patient a transient ischemic attack led to a hospitalization after 10 days on varenicline. The patient showed a transient hemiparesis on the left side. Symptoms dissolved within hours. The patient previously has had a stroke. The same patient reported mild hallucinations after increasing varenicline dose to 1 mg twice daily. As the patient was further interested in pharmacological support, dosage was reduced but symptoms reoccurred, and the patient decided to switch to nicotine replacement therapy.

One patient receiving varenicline, who stopped the medication due to nausea after 4 weeks, suffered renal failure about 3 weeks later due to chemotherapy-associated diarrhea. The patient finally died due to renal failure.

One patient receiving varenicline died by suicide after receiving the message of progress of lung cancer. The patient was a hunter. He received only one package of varenicline, which was taken until 4 weeks before his dead. The patient never received a further package of varenicline. There was no suggestion that the medication was responsible for his decision to commit suicide.

## Discussion

This prospective study evaluated a comprehensive smoking cessation program including pharmacotherapy in smokers with newly diagnosed lung cancer. Achieved smoking abstinence rate was 37.5% after 12 and 32.8% after 24 weeks and thus comparable to results in other patient groups or healthy subjects [15, 16, 24]. A recent unblinded study randomly assigned 303 patients with newly diagnosed cancer to an intensive treatment group, with 11 smoking cessation telephone counselling sessions coupled with 12 weeks of free cessation medication or to standard therapy [25]. The 7-day abstinence rates at 6-month were 35% in the intensive treatment versus 22% in the standard group. A study using only brief advice in cancer patients reported a biochemically validated quit rate at 6 months of 6% [26]. When comparing these and other studies, it should be kept in mind that comparison is difficult because of diversities in enrolled patients, assessment of abstinence and intensity of the cessation intervention.

There is only limited evidence on smoking cessation in lung cancer patients. Previous studies were retrospective [27] and included only a limited number of patients (15–49 patients) [9, 10, 28]. Importantly, side effects of pharmacotherapy were not systematically evaluated. Thus, a recent Cochrane review stated insufficient evidence for smoking cessation in patients with lung cancer mainly due to the limited number of patients with lung cancer included and the uncontrolled design [8].

Smoking abstinence was numerically higher in the varenicline group as compared to the nicotine replacement group, but this observational study was not designed to show differences between different forms of treatment. In persons trying to quit smoking varenicline is known to be more effective as compared to nicotine replacement therapy [16, 29].

Most of the patients chose pharmacotherapy following smoking cessation counselling. Only six patients decided against any medication. Among these, most patients had already stopped smoking within the 4 weeks before enrollment into the study and the score of the Fagerström-test was lower than in the other patients, indicating lesser nicotine addiction. Thus, our study revealed that intense cessation counselling during hospitalization for diagnosis and subsequent treatment of lung cancer leads to a high proportion of patients using pharmacotherapy as an aid for smoking cessation. In a prospective, representative German survey of over 10.000 smokers, cessation attempts only 7.6% used nicotine replacement therapy and 0.4% used varenicline [30]. A high proportion of patients using pharmacotherapy is important since this nearly doubles the success rate [31].

We included not only current smokers but also patients smoking up to 4 weeks before study enrollment. This was done because quitting just before the diagnosis of lung cancer is common [32], often due to acute lung cancer associated symptoms and may thus indicate a less favorable abstinence rate [33]. Accordingly, cessation intervention is endorsed in these patients [34].

### Side effects

Side effects of medication are important when treating lung cancer patients with a high symptom burden and a limited prognosis. The side effects observed in the present study were keeping within the previously published data in persons trying to quit smoking. There were no solicited adverse events reported in patients using nicotine replacement therapy. Nausea while taking varenicline was reported by six patients. Of note, all of these patients received concurrent chemotherapy or radiochemotherapy, respectively. There was no difference in the subscale “nausea and vomiting” on the EORTC QLQ C30 questionnaire between the patients receiving varenicline or nicotine replacement therapy. Also, in these groups nausea and vomiting were associated with lung cancer therapy. Thus, the study revealed no novel side effects in the cohort of patients with life-limiting disease often undergoing systemic therapy including chemotherapy and immunotherapy. As the study included patients with all lung cancer stages, among them many patients with advanced disease, most patients who died did so due to progression of lung cancer. Accordingly, mortality was high during the study period.

Symptoms of nicotine withdrawal were lowest in patients who did not opt for additional pharmacological treatment. This might be explained by the low rate of current smokers in this group at study entry as discussed above. Correspondingly, the abstinence rate was highest in this group (60% of patients were abstinent at week 12). The significant difference of nicotine withdrawal symptoms at week 12 between patients receiving NRT or varenicline and patients without medication might also be explained by the disparity of the groups at baseline. The small number of patients in this group hampers interpretation of the data. There were no significant group differences for global health status, and cognitive as well as emotional functioning.

### Limitations

A limitation of the present study is that not all smokers with a new diagnosis of lung cancer were included in the study since some patients were reluctant to participate and the study team was absent for distinct periods of time. Furthermore, the study is rather small and the lack of randomization precludes any formal comparison between the different pharmacologic treatments. Finally, qualitative data from patients and clinicians on their experiences might have helped to identify barriers. A strength of the study is the prospective evaluation of side effects and the real life setting in a tertiary lung cancer center.

## Conclusion

Our results indicate that smoking cessation is feasible in patients with newly diagnosed lung cancer. The observed cessation rate is comparable to other patient cohorts or healthy subjects. Furthermore, pharmacotherapy using nicotine replacement therapy or varenicline in addition to cancer therapy was safe and did not show novel side effects in these seriously ill patients. Given the known positive effects of smoking cessation in lung cancer patients, smoking cessation should be integral part of lung cancer treatment [35].

## Supplementary Information

**Additional file 1.** Comparison of Mean Total HADS during treatment period.

**Additional file 2.** Comparison of Mean EORTC-QLQ C30 3.0 subscale nausea and vomiting. Comparison of Mean EORTC-QLQ C30 3.0 subscale fatigue and insomnia.

## Data Availability

The datasets used and analysed during the current study is available from the corresponding author on reasonable request.

## Abbreviations

CO: Carbon monoxide
BfArM: Bundesinstitut für Arzneimittel und Medizinprodukte
EORTC QLQ: European Organization Research Treatment Cancer—Quality of Life Questionnaire
EQ VAS: European Quality of Life—visual analog scale
GCP: Good clinical practice
HADS: Hospital Anxiety and Depression Scale
MPSS: Mood and Physical Symptoms Scale
NM: No medication
NRT: Nicotine replacement therapy
STD: Standard deviation
