# Supplementary material for: Smoking cessation by combined medication and counselling: a feasibility study in lung cancer patients
Source: BMC Pulm Med. 2022 Jun 27;22:252. doi: 10.1186/s12890-022-02048-1 (PMC9235273; doi:10.1186/s12890-022-02048-1)
Supplement: Supplementary file 1 — Additional file 1. Comparison of Mean Total HADS during treatment period. [file 12890_2022_2048_MOESM1_ESM.docx]

Supplement table HADS: Comparison of Mean Total HADS during treatment period.

| **Visite** | **Contrast** | **Method** | **t Value** | **DF** | **Mean** | **LCL** | **UCL** | **p-Value** |
| --- | --- | --- | --- | --- | --- | --- | --- | --- |
| Day 1 | NRT vs. Varenicline | Pooled | -0.95 | 69 | -1.51 | -4.68 | 1.67 | 0.3471 |
| Day 1 | NRT vs. NM | Pooled | 0.35 | 40 | 0.92 | -4.43 | 6.26 | 0.7306 |
| Day 1 | Varenicline vs. NM | Pooled | 0.73 | 39 | 2.42 | -4.27 | 9.12 | 0.4683 |
| Week 6 | NRT vs. Varenicline | Pooled | -0.10 | 59 | -0.16 | -3.33 | 3.02 | 0.9210 |
| Week 6 | NRT vs. NM | Pooled | 1.62 | 35 | 5.23 | -1.32 | 11.79 | 0.1138 |
| Week 6 | Varenicline vs. NM | Pooled | 1.64 | 30 | 5.39 | -1.32 | 12.11 | 0.1114 |
| Week 12 | NRT vs. Varenicline | Pooled | -0.57 | 44 | -1.17 | -5.35 | 3.01 | 0.5743 |
| Week 12 | NRT vs. NM | Pooled | 2.01 | 26 | 6.27 | -0.13 | 12.67 | 0.0544 |
| Week 12 | Varenicline vs. NM | Pooled | 1.97 | 26 | 7.44 | -0.33 | 15.21 | 0.0597 |

NRT = Nicotine replacement therapy. NM = no medication.
