# Supplementary material for: Smoking cessation by combined medication and counselling: a feasibility study in lung cancer patients
Source: BMC Pulm Med. 2022 Jun 27;22:252. doi: 10.1186/s12890-022-02048-1 (PMC9235273; doi:10.1186/s12890-022-02048-1)
Supplement: Supplementary file 2 — Additional file 2. Comparison of Mean EORTC-QLQ C30 3.0 subscale nausea and vomiting. Comparison of Mean EORTC-QLQ C30 3.0 subscale fatigue and insomnia. [file 12890_2022_2048_MOESM2_ESM.docx]

Supplement table - EORTC

Comparison of Mean EORTC-QLQ C30 3.0 subscale nausea and vomiting

|  | **Treatment** | **Method** | **t Value** |  | **DF** | **Mean** | **LCL** | **UCL** | **p-Value** |
| --- | --- | --- | --- | --- | --- | --- | --- | --- | --- |
| Day 1 | NRT vs. Varenicline | Pooled | -0.24 |  | 59.054 | -0.81 | -7.58 | 5.96 | 0.8115 |
| Day 1 | NRT vs. NM | Pooled | 0.65 |  | 41 | 3.08 | -6.53 | 12.69 | 0.5215 |
| Day 1 | Varenicline vs. NM | Pooled | 0.56 |  | 39 | 3.89 | -10.27 | 18.05 | 0.5817 |
| Week 12 | NRT vs. Varenicline | Pooled | 0.21 |  | 45 | 1.72 | -14.50 | 17.94 | 0.8317 |
| Week 12 | NRT vs. NM | Pooled | 1.11 |  | 26 | 14.64 | -12.49 | 41.76 | 0.2775 |
| Week 12 | Vareniclin vs. NM | Pooled | 0.97 |  | 27 | 12.92 | -14.31 | 40.14 | 0.3390 |

subscale *nausea and vomiting* at day 1 and week 12. NRT = Nicotine replacement therapy. NM = no medication

Comparison of Mean EORTC-QLQ C30 3.0 subscale fatigue and insomnia

|  | **Treatment** | **Method** | **t Value** | **DF** | **Mean** | **LCL** | **UCL** | **p-Value** |
| --- | --- | --- | --- | --- | --- | --- | --- | --- |
| Day 1 | NRT vs. Varenicline | Pooled | -1.63 | 68 | -13.56 | -30.18 | 3.05 | 0.1080 |
| Day 1 | NRT vs. NM | Pooled | -1.29 | 40 | -19.44 | -49.98 | 11.09 | 0.2054 |
| Day 1 | Varenicline vs. NM | Pooled | -0.36 | 38 | -5.88 | -39.09 | 27.33 | 0.7219 |
| Week 12 | NRT vs. Varenicline | Pooled | -0.25 | 45 | -2.48 | -22.47 | 17.52 | 0.8042 |
| Week 12 | NRT vs. NM | Pooled | 1.57 | 26 | 27.25 | -8.53 | 63.02 | 0.1296 |
| Week 12 | Vareniclin vs. NM | Pooled | 1.92 | 27 | 29.72 | -2.08 | 61.53 | 0.0658 |

subscale *insomnia* at day 1 and week 12. NRT = Nicotine replacement therapy. NM = no medication
